# Patient safety culture in private hospitals in China: a cross-sectional study using the revised Hospital Survey on Patient Safety Culture

**DOI:** 10.3389/fpubh.2024.1323716

**Published:** 2024-06-05

**Authors:** Yan Liu, Jianing Xu, Xiaoguang Yang, Liu Yue, Guohong Li, Alastair P. Mah

**Affiliations:** ^1^Department of Quality and Safety, United Family Healthcare, Beijing, China; ^2^School of Public Health, Shanghai Jiao Tong University School of Medicine, Shanghai, China; ^3^China Hospital Development Institute, Shanghai Jiao Tong University School of Medicine, Shanghai, China; ^4^Department of Quality and Safety, Beijing United Family Hospital, United Family Healthcare, Beijing, China; ^5^Department of Medical Affair, United Family Healthcare, Beijing, China; ^6^Faculty of Health, School of Medicine, Deakin University, Geelong, VIC, Australia; ^7^Faculty of Medicine, The Chinese University of Hong Kong, Hong Kong SAR, China

**Keywords:** patient safety culture, adverse events, HSOPSC 2.0, private hospital, China

## Abstract

**Background:**

This study aimed to translate the revised Hospital Survey on Patient Safety Culture (HSOPSC 2.0) to Mandarin, evaluate its psychometric properties, and apply it to a group of private hospitals in China to identify the determinants associated with patient safety culture.

**Methods:**

A two-phase study was conducted to translate and evaluate the HSOPSC 2.0. A cross-cultural adaptation of the HSOPSC 2.0 was performed in Mandarin and applied in a cross-sectional study in China. This study was conducted among 3,062 respondents from nine private hospitals and 11 clinics across six cities in China. The HSOPSC 2.0 was used to assess patient safety culture. Primary outcomes were measured by the overall patient safety grade and patient safety events reported.

**Results:**

Confirmatory factor analysis results and internal consistency reliability were acceptable for the translated HOSPSC 2.0. The dimension with the highest positive response was “Organizational learning - Continuous improvement” (89%), and the lowest was “Reporting patient safety event” (51%). Nurses and long working time in the hospital were associated with lower assessments of overall patient safety grades. Respondents who had direct contact with patients, had long working times in the hospital, and had long working hours per week reported more patient safety events. A higher level of patient safety culture implies an increased probability of a high overall patient safety grade and the number of patient safety events reported.

**Conclusion:**

The Chinese version of HSOPSC 2.0 is a reliable instrument for measuring patient safety culture in private hospitals in China. Organizational culture is the foundation of patient safety and can promote the development of a positive safety culture in private hospitals in China.

## 1 Introduction

Patient safety is the foremost priority of global health. It is the fundamental requirement for medical care and one of the key domains of quality management at all levels of healthcare (1). Over 1 in 10 patients continue to be harmed from safety lapses during their care. Globally, unsafe care results in well over 3 million deaths each year. In the developing world, as many as 4 in 100 people die from unsafe care (2, 3). One identified reason for such unsafe patient care is a weak safety culture. The shared attitudes, beliefs, and values of all employees lead to behavioral norms in the organization. These norms create a cultural climate of patient safety that promotes consistent performance for patient safety, which is associated with improved patient outcomes (4). As an integral component of healthcare quality management, a positive patient safety culture improves the attitudes and perceptions of patient safety at an individual level, reduces the occurrence of adverse events, and improves the overall safety of the healthcare delivery system (5, 6).

Measurement of patient safety culture in China is limited and has only been conducted in a few public hospitals and in almost no private hospitals (7). Since 2011, China’s healthcare reform has emphasized private investment in healthcare services, and driven by domestic demand and policy, the number of private hospitals has risen from 30.8% of the total number of hospitals in China in 2009 to 67.7% in 2021 (8). Private hospitals have gradually become an important part of China’s healthcare system to meet diverse healthcare needs.

The Hospital Survey on Patient Safety Culture (HSOPSC), developed by the Agency for Healthcare Research and Quality (AHRQ), is the most widely used instrument to measure safety culture in healthcare organizations internationally. In 2019, AHRQ released a revised version, HSOPSC 2.0, and encouraged its use in place of the original version (9). A previous study contributed to the availability of a Chinese version of the revised surveys on patient safety culture, but it focus on the public hospital nursing team (10). Considering that public and private hospitals setting is very different, and private hospitals constitute a larger proportion of registered hospitals in China currently. Therefore, this study translated the HSOPSC 2.0 to Mandarin and validated it for use in private hospitals in China, also captures a wider breadth of professions instead of a single discipline. The purpose was to identify strengths and areas for improvement related to organizational culture and patient safety by measuring the perception of patient safety culture and identifying the factors associated with overall patient safety grade and adverse events reporting. The study results could provide a reference for managers and policymakers to promote the construction of a patient safety culture in private healthcare institutions.

## 2 Materials and methods

### 2.1 Study design and setting

A two-phase practice was conducted to translate and evaluate the HSOPSC 2.0. The original English version was translated into Mandarin and adapted following published recommendations (11). Each item was assessed for clarity and cultural relevance and then evaluated for internal consistency and construct validity. Subsequently, a descriptive cross-sectional study was conducted using the bilingual version.

This cross-sectional study was conducted in nine private hospitals and 11 clinics across six cities in China from 7 to 28 February 2022. The sampling method used in this study was convenience sampling; all the hospitals and clinics are within a group network. An online survey was used to avoid personal contact due to the Coronavirus disease 2019 (COVID-19) pandemic. The survey was strictly anonymous to ensure the privacy of the respondents. All participants were informed about the intention of the study. Two blind researchers were previously instructed about the questionnaire’s content and provided with specific training on quality control. They addressed questions and clarified doubts if participants did not understand the questionnaire.

Efforts were made to reduce nonresponse rates and mitigate potential biases introduced by non-respondents. Reminders through emails and instant messaging software were send to participants on a weekly basis, and a lucky draw was conducted at the end, encouraging completion of the questionnaire. Furthermore, the survey was designed to be strictly anonymous, to ensure participant privacy and reduce social desirability bias. Participants were assured that their responses would remain confidential, which encouraged more honest and representative participation.

Ethical clearance and approval was obtained from the Ethics Committee of Beijing United Family Hospital. Considering that this survey is purely anonymous and does not involve the personal information of participants, the committee approved that participants do not need to sign the informed consent and can be informed the intent of the study at the beginning of the questionnaire.

### 2.2 Instrument

Patient safety culture is conceptually complex and can be viewed within the Patient Safety Culture Theoretical Framework, which is made up of these components: (a) degree of psychological safety, (b) degree of organizational culture, (c) quality of culture of safety, (d) degree of high reliability organization, (e) degree of deference to expertise, and (f) extent of resilience (12).

To measure this complex concept, one commonly used instrument is the Agency for Healthcare Research and Quality Hospital Survey on Patient Safety Culture. The HSOPSC 2.0 questionnaire was developed in 2019 and used in this study to assess hospital staff’s perceptions of patient safety culture. The latest version, 2.0, reduced the number of survey items from 51 to 40 and dropped the dimensions from 12 to 10. Some items were reworded because they were sensitive, semantically redundant, or difficult to translate (11). “Does not apply/Do not know” response option was added to each item. Meanwhile, HSOPSC 2.0 changed the response options from ‘failing’ to ‘poor’ and ‘acceptable’ to ‘good’ in overall patient safety grade.

The questionnaire was translated into Mandarin and modified to fit the Chinese hospital setting. Two independent translators did the preliminary English-to-Chinese translation. Each preliminary version was blindly back-translated by two other people. All translators were bilingual and with expertise in health, including employees at the World Health Organization, physicians in tertiary hospitals, and students at the University of Virginia School of Medicine. The preliminary version was then evaluated by a translation committee of five bilingual medical professors and synthesized into the latest version.

### 2.3 Participants and data collection

This study recruited 3,881 hospital staff, including physicians, nurses, technicians, and administrators. A total of 3,064 participants completed the online survey, giving a response rate of 78.95%. The responses were examined for incomplete or invalid data. The exclusion criteria were as follows: (1) surveys were completely blank; (2) contained “Does not apply/Do not know” responses for all survey items; or (3) contained the same answer for all the items. Two invalid questionnaires were eventually excluded, leaving 3,062 questionnaires for analysis.

### 2.4 Statistical analysis

The internal consistency of the translated HSOPSC 2.0 was assessed using Cronbach’s α coefficient, with a value of 0.7 considered acceptable. The appropriateness of the factor analysis was evaluated using the Kaiser-Meyer-Olkin (KMO) test (>0.5) and Bartlett’s test of sphericity (*p* < 0.05). Confirmatory factor analysis (CFA) was performed using AMOS version 24 to confirm the factor structure of the questionnaire. As recommended by Jackson (13), we evaluated the following goodness-of-fit indices of the measurement model: Chi-square goodness of fit (*p* > 0.05), root mean square error of approximation (RMSEA, < 0.08), standardized root mean residual (SRMR, < 0.05), and comparative fit index (CFI, > 0.9).

Categorical variables were presented as frequencies with percentages. Differences in categorical outcomes were assessed using the Chi-square test. Percentage of positive responses for each item and dimension were calculated. Responses of ‘agree’ or ‘strongly agree’ and ‘always’ or ‘most of the time’ for the positively worded items indicated positive responses. Additionally, responses of ‘disagree’ or ‘strongly disagree’ and ‘never’ or ‘rarely’ for the negatively worded items indicated positive responses. Positive response rates were used to evaluate attitudes toward patient safety culture in different dimensions. A positive response rate > 75% indicated a strong area of safety culture, while <50% needed improvement (14).

The relationship between the explanatory variables (demographic characteristics and 10 dimensions of patient safety culture) and the outcome variables (overall patient safety grade and the number of patient safety events reported) was examined using binary logistic regression. The outcome variable was dichotomized into high (‘excellent’ and ‘very good’) and low (‘poor’ to ‘good’) overall patient safety grades, and ‘none’ and ‘1 or more’ reported patient safety events. We treated staff position, duration working for the hospital, working hours per week, and contact with patients as dummy variables and used a forward stepwise logistic regression approach. Multicollinearity in the logistic model was checked using the variance inflation factor (VIF < 10). The goodness-of-fit of the models was assessed using the Hosmer-Lemeshow test (*p* > 0.05). All statistics were managed with Office Excel 2010 and analyzed using IBM SPSS version 24.0. Two-sided *p*-values <0.05 were considered significant.

## 3 Results

### 3.1 Characteristics of respondents

A total of 3,062 respondents from nine hospitals and 11 clinics in six cities across China completed the survey. The mean age of the respondents was 41 (SD 10) years old, and most were female (79.5%). The majority of the respondents (72.8%) had at least an undergraduate degree, 523 (17.1%) were physicians, 1,002 (32.7%) were nurses, and 911 (29.8%) were administrative staff. Of the respondents, 39.3% had worked in the hospital for 1–5 years, while 27.12% worked for 6–10 years. Half of respondents (50.2%) worked 30–40 h a week. Additionally, 74.8% of respondents had direct contact with patients.

### 3.2 Dimensionality, reliability and validity of the instrument

The dimensionality of the translated HSOPSC 2.0 was assessed using confirmatory factor analysis. The results supported the proposed factor structure, indicating that the instrument captured the intended dimensions of patient safety culture in the context of private hospitals in China.

The HSOPSC 2.0 has been widely used and validated in various countries. The Cronbach’s α for the 10 subscales ranged from 0.67 to 0.89 in the U.S. study and 0.61 to 0.83 in the Korean study (11, 15), which provides evidence of internal consistency reliability. In this study, the overall Cronbach’s α of the HSOPSC 2.0 was 0.91, and Cronbach’s α coefficients of the subscales ranged from 0.52 to 0.88, slightly lower than the other study, which still implied acceptable reliability.

Bartlett’s test demonstrated a sufficient inter-item correlation (*p* < 0.001), and the KMO test (0.92) indicated a high model adequacy. The Chi-square test was statistically significant: *χ*^2^/df ratio = 8.77 (*χ*^2^ = 3664.95, df = 418, *p* < 0.001), possibly due to the large sample size. The other multiple indices indicated that the ten-factor model provided a good fit to the data: RMSEA = 0.05 (<0.08), SRMR = 0.048 (<0.05), and CFI = 0.91 (> 0.9).

These findings indicate that the adapted survey instrument, the Mandarin version of HSOPSC 2.0, is valid and reliable for measuring patient safety culture in private hospitals in China.

### 3.3 HSOPSC 2.0 score

Among these 10 safety culture dimensions, seven were strength areas with over 75% positive response rate. The other three dimensions ranged from 51 to 73%. There were no dimensions with a positive response rate below 50%, indicating a need for improvement. In this study, the highest positive response rate dimension was “Organizational learning - Continuous improvement” (89%), and the lowest was “Reporting patient safety event” (51%). Table 1 shows the average positive response rates of composite measures for this study were higher than the 2021 U.S. database report (16).

**Table 1:** Scores of the HSOPSC 2.0 and each dimension.

Safety culture dimensions	Average positive response rate (%)			
	This study	Rank	2021 U.S.*	Rank
Organizational learning - continuous improvement	89	1	72	5
Teamwork	88	2	82	1
Supervisor, manager, or clinical leader support for patient safety	86	3	80	2
Handoffs and information exchange	83	4	64	8
Hospital management support for patient safety	82	5	67	7
Communication about error	77	6	64	8
Communication openness	77	7	75	3
Communication about error	73	8	71	6
Staffing and work pace	55	9	58	10
Reporting patient safety event	51	10	74	4
Composite measures average	76		71	

^*^U.S. Positive response rate published by the AHRQ.

Most participants (65%) rated their unit “excellent” (27%) or “very good” (38%) in patient safety, which was slightly lower than the U.S. rate (69%). Less than half of the respondents (44%) reported at least one event in their hospital in the past year, similar to the U.S. report (46%).

### 3.4 Univariate analysis of factors correlated with patient safety grade and patient safety events reported

As shown in Table 2, staff position, working time in the hospital, and contact with patients contributed to significant differences in the overall patient safety grade and the number of events reported (*p* < 0.05). Moreover, working hours per week led to significant differences in the reported number of events (*p* < 0.05).

**Table 2:** Univariate analysis results.

	Overall patient safety grade (*N* = 3,062)		Patient safety events reported (*N* = 3,062)			
	High	Low	*p* value	None	1 or more	*p* value
Region			0.763			0.731
North China	1,033(52.0)	560(52.1)		884(51.8)	709(52.3)	
East China	750(37.7)	412(38.4)		656(38.5)	506(37.3)	
South China	205(10.3)	102(9.5)		166(9.7)	141(10.4)	
Staff position			<0.001			<0.001
Physicians	333(16.8)	190(17.7)		295(17.3)	228(16.8)	
Nurses	563(28.3)	439(40.9)		493(28.9)	509(37.5)	
Administrator	647(32.5)	264(24.6)		554(32.5)	357(26.3)	
Support	231(11.6)	95(8.8)		219(12.8)	107(7.9)	
Other clinical position	197(9.9)	80(7.4)		125(7.3)	152(11.2)	
Others	17(0.9)	6(0.6)		20(1.2)	3(0.2)	
Working time in the hospital (year)			<0.001			<0.001
< 1	486(24.4)	193(18.0)		504(29.5)	175(12.9)	
1–5	802(40.3)	400(37.2)		643(37.7)	559(41.2)	
6–10	483(24.3)	347(32.3)		390(22.9)	440(32.4)	
≥ 11	217(10.9)	134(12.5)		169(9.9)	182(13.4)	
Working hours per week (hour)			0.554			<0.001
< 30	52(2.6)	23(2.1)		54(3.2)	21(1.5)	
30–40	1,005(50.6)	531(49.4)		888(52.1)	648(47.8)	
> 40	931(46.8)	520(48.4)		764(44.8)	687(50.7)	
Contact with patients			<0.001			<0.001
Yes	1,446(72.7)	843(78.5)		1,170(68.6)	1,119(82.5)	
No	542(27.3)	231(21.5)		536(31.4)	237(17.5)	

### 3.5 Binary logistic regression analysis for patient safety grade and patient safety events reported

Regarding the overall patient safety grade, the model was well calibrated (Hosmer-Lemeshow test, *p* = 0.992), and there was no multicollinearity problem (all VIF < 10). The binary logistic regression showed that only staff position and working time in the hospital were influencing factors. Nurses and long working time in the hospital were associated with lower assessments of overall patient safety grades. The binary analysis showed that seven dimensions of patient safety culture were significantly associated with overall patient safety grade (Table 3). A higher level of patient safety culture indicates an increased probability of a high overall patient safety grade.

**Table 3:** Binary logistic regression models with overall patient safety grade.

	OR(95%CI)	*p* value
Staff position		
Physicians (reference)		
Nurses	1.27(1.00–1.62)	0.052
Administrator	0.61(0.47–0.79)	<0.001
Support	0.49(0.35–0.69)	<0.001
Other clinical position	0.66(0.46–0.93)	0.017
Others	0.40(0.14–1.17)	0.093
Working time in this hospital (years)		
< 1 (reference)		
1–5	1.27(1.01–1.59)	0.039
6–10	1.86(1.46–2.37)	<0.001
≥ 11	1.73(1.27–2.35)	<0.001
Teamwork	0.74(0.64–0.86)	<0.001
Staffing and work pace	0.87(0.81–0.94)	<0.001
Response to error	0.86(0.80–0.94)	0.001
Supervisor, manager, or clinical leader support for patient safety	0.87(0.77–0.99)	0.027
Communication openness	0.83(0.78–0.90)	<0.001
Reporting patient safety event	0.85(0.76–0.94)	0.002
Hospital management support for patient safety	0.68(0.61–0.75)	<0.001

Regarding the number of reported patient safety events, the model is well calibrated (Hosmer-Lemeshow test, *p* = 0.916), and there is no multicollinearity problem (all VIF < 10). The respondents who had contact with patients, long working time in the hospital, and long working hours per week reported more patient safety events. Nurses, administrators, support, and other clinical positions reported more patient safety events than physicians. Furthermore, Table 4 shows that the incidence of patient safety events reported was closely related to higher levels of patient safety culture.

**Table 4:** Binary logistic regression models with patient safety events reported.

	OR(95%CI)	*p* value
Region		
North China (reference)		
East China	1.07(0.90–1.26)	0.459
South China	1.47(1.12–1.93)	0.006
Staff position		
Physicians (reference)		
Nurses	1.35(1.07–1.70)	0.011
Administrators	1.13(0.88–1.45)	0.330
Support	1.47(1.03–2.09)	0.032
Other clinical position	1.87(1.36–2.57)	<0.001
Others	0.34(0.09–1.21)	0.095
Working time in the hospital (year)		
< 1 (reference)		
1–5	2.42(1.95–3.01)	<0.001
6–10	3.14(2.49–3.97)	<0.001
≥ 11	2.95(2.21–3.95)	<0.001
Working hours per week (hour)		
< 30	0.53(0.31–0.92)	0.024
30–40	0.78(0.67–0.92)	0.002
> 40 (reference)		
Contact with patients		
Yes	1.76(1.41–2.20)	<0.001
No (reference)		
Communication about error	1.12(1.01–1.23)	0.029
Communication openness	1.08(1.01–1.15)	0.036
Reporting patient safety event	1.47(1.33–1.62)	<0.001
Hospital management support for patient safety	0.79(0.71–0.87)	<0.001
Handoffs and information exchange	1.14(1.05–1.24)	0.001

## 4 Discussion

### 4.1 Statement of principal findings

Medical practice is a complex domain with high risks, uncertainties, and layered dynamics. Developing a safety culture is a cost-effective strategy for building a safer healthcare system (17). Efforts to promote a safety culture are associated with better patient outcomes, improved efficiency, and fewer adverse events (1). However, few studies address patient safety as a health strategy for strengthening the private hospital system. This study is the first of its kind in China to explore safety culture issues and the influencing factors in private hospitals.

The overall average positive response rate (76%) was higher than studies conducted in South Korean hospitals (43%) and American hospitals (71%), indicating higher levels of patient safety culture among the staff in this study (13, 15). Among the 10 dimensions of patient safety culture, “Organizational learning - Continuous improvement” was the highest contributing dimension for overall patient safety culture. This implies that the sampled hospitals paid more attention to patient safety issues by providing resources to support patient safety matters and making continuous improvements. Recently, more hospitals in China have actively created an organizational atmosphere of learning while working. Creating a culture of learning and sustainable development is linked to both individual capacity building and organizational performance, and it is considered to be an important factor in facilitating safer and more efficient healthcare delivery (18). Furthermore, “Teamwork” has emerged as one of the top two highest positive response rate dimensions in almost all HSOPC studies, especially in China (19, 20). Previous studies also found that Chinese have a greater appreciation for collectivism (21). This might be associated with Chinese tradition, which encourages collectivist theories and places relatively more emphasis on cooperation.

### 4.2 Interpretation within the context of the wider literature

This study demonstrated that the areas with the most potential for improvement were “Staffing and work pace” and “Reporting patient safety event.” The relatively low positive response rate for “Staffing and work pace” is similar to previous studies in South Korea (15). According to the Organization for Economic Co-operation and Development (OECD), there were 3.1 nurses and 2.2 doctors per 1,000 population in China, 7.9 nurses and 2.5 doctors per 1,000 population in Korea, which were both well below the average level of about 8.8 nurses and 3.6 doctors (22). Chinese medical staff work in a challenging environment with staff shortages and heavy workloads, which could potentially contribute to clinician burnout and increase the risk of patient safety events (23). One possible explanation is that health workers face a greater workload and intensity because of the COVID-19 pandemic. One of the continuing negative effects of the increase in patient numbers and care intensity is staff shortages. Therefore, the government should take measures to increase the number of medical staff and rationally plan and allocate medical resources.

From 2007 to 2022, seven versions of the Patient Safety Goal were released by the Chinese Hospital Association, each of which included the goal of encouraging medical staff to report patient safety events voluntarily (7). Although there is a consensus that physicians are important in patient safety, this study found that they were less likely to report events, consistent with previous findings in China (24). Compared with physicians, nurses spend more time communicating with patients, giving them more opportunities to identify and report patient safety concerns. Likewise, administrators reported more events, possibly because they placed more emphasis on patient safety or had easier access to the reporting system. A surprising finding of this study is that longer years of service were associated with higher reporting of patient safety events and, by contrast, lower overall perception of safety grades. A possible explanation is that as seniority increases, the experiences, social interactions, perceptions, and values related to patient safety become more complex. Senior medical staff resuscitate acute critical patients, are exposed to higher medical risks, are more aware of the safety practices and benefits of reporting conducted within the hospital, and are more concerned about patient safety (25). This indicates that a culture of improvement is as important to patient safety as a culture of reporting.

Chegini et al. (26) demonstrated that the number of events reported in private hospitals was higher than in public hospitals, which may be the different safety-relevant interventions related to reporting, analysis, and prevention of adverse events in the public and private sectors. Private hospitals are more concerned with a patient-safety-oriented management approach to improve the quality and safety of care (27). In the univariate analysis, employees with less than 1 year of service reported fewer patient safety events than employees with 1–5 years and 6–10 years of service. This may be linked to organizational culture, where individuals’ perceptions converge as their time in the organization increases. Thus, organizational culture is fundamental to patient safety.

### 4.3 Strengths and limitations

This study has several strengths, including that it is the first study in China to explore patient safety culture issues and its influencing factors in private hospitals. Additionally, HSOPSC 2.0 was translated and tested for application in China, which provides a reference for safety culture assessment. Moreover, some influencing factors related to patient safety culture were identified. Nevertheless, some limitations should be considered in this study despite its strengths. The Cronbach’s α in some dimensions was lower than 0.7, which might be due to cultural differences between China and the U.S. Furthermore, the generalizability of the findings is not sufficiently clear due to the convenience method, the potential for nonresponse bias in the online survey, and the absence of thorough discussion on confounding factors. However, the response rate was actually high so largely mitigated. Moreover, data for this study were collected exclusively from private hospitals, and all sampled hospitals are part of a large healthcare organization, self-reported data may introduce biases. Further research should be undertaken to extend the scope and sample size as well as compare public and other private hospitals in China.

### 4.4 Implications for policy, practice, and research

It is important to acknowledge that patient safety depends on a systems approach, which requires contributions and collaboration from various stakeholders. The results of this study suggest that hospitals and healthcare organizations should have imperatives to (1) establish a non-punitive, high-security, and voluntary reporting culture; (2) rationally allocate human resources and work intensity to focus on insecurity and dysphoria among nurses and medical staff; (3) establish a culture of improvement to promote positive feedback on reporting; and (4) integrate patient safety education into teaching curriculum and clinical practice to establish an organizational culture. These will be important strategies with far-reaching applicability in ensuring quality care and patient safety.

## 5 Conclusion

As far as the authors are aware, this is the first study conducted in China to validate HSOPSC 2.0 and evaluate patient safety culture in private hospitals. Developing and maintaining a positive patient safety culture among healthcare staff is widely acknowledged as crucial to improving patient safety in healthcare organizations. HSOPSC 2.0 had satisfactory reliability and validity to be applied in private hospitals in China. Organizational culture can promote patient safety and facilitate the development of a positive safety culture in private hospitals in China. The results of this study provide some evidence for developing effective strategies to promote safety culture to ensure patient safety and quality of care.

## Data availability statement

The raw data supporting the conclusions of this article will be made available by the authors, without undue reservation.

## Author contributions

YL: Writing – review & editing, Writing – original draft, Validation, Supervision, Software, Project administration, Methodology, Investigation, Formal analysis, Data curation, Conceptualization. JX: Writing – original draft, Validation, Software, Methodology, Formal analysis, Data curation. XY: Writing – original draft, Resources, Methodology, Investigation, Data curation. LY: Writing – original draft, Validation, Resources, Project administration, Methodology. GL: Writing – review & editing, Supervision, Resources, Methodology, Conceptualization. AM: Writing – review & editing, Visualization, Supervision, Resources, Project administration, Methodology, Conceptualization.
